# Machine Learning Applications for Risk Stratification in Heart Failure with Preserved Ejection Fraction: A New Era in Cardiology

**DOI:** 10.3390/diagnostics16101545

**Published:** 2026-05-19

**Authors:** Bodour S. Rajab

**Affiliations:** Department of Clinical Laboratory Sciences, Faculty of Applied Medical Sciences, Umm Al-Qura University, Makkah 21955, Saudi Arabia; bsrajab@uqu.edu.sa

**Keywords:** heart failure, machine learning, risk prediction, phenotyping, precision care

## Abstract

Heart failure with preserved ejection fraction (HFpEF) is a prevalent and heterogeneous syndrome with limited therapeutic options, making accurate risk stratification essential yet challenging. Traditional tools such as the H2FPEF and HFA-PEFF scores incorporate few variables and demonstrate modest prognostic performance. Machine learning (ML) offers enhanced risk prediction by integrating multidimensional clinical, imaging, biomarker, and molecular data. This review summarizes current ML applications in HFpEF, including random forests, gradient boosting, support vector machines, and deep learning, highlighting their superior discrimination and ability to reveal phenotypic subgroups with distinct outcomes. We also address practical considerations such as interpretability, real-world validation, and integration into clinical workflows, as well as challenges related to data bias, generalizability, and regulatory requirements. Future opportunities include real-time clinical decision support, digital health integration, and interventional ML to guide personalized therapy. ML holds significant potential to advance precision care and improve outcomes in HFpEF.

## 1. Introduction

Heart failure with preserved ejection fraction (HFpEF) constitutes a substantial and escalating challenge in cardiovascular therapy, comprising over 50% of all heart failure (HF) cases globally and a continuously rising prevalence [1]. For context, heart failure is classified by LVEF: HFrEF is defined as LVEF ≤ 40%, characterized by systolic dysfunction with well-established guideline-directed therapies; HF with mildly reduced ejection fraction (HFmrEF) includes LVEF 41–49%, representing an intermediate phenotype; and HFpEF (LVEF ≥ 50%) lacks effective therapies, exhibits marked heterogeneity, and presents the greatest challenge for risk stratification [1,2]. On the other hand, HFpEF poses significant diagnostic and treatment challenges given its diverse presentation and the absence of a clear diagnostic signature, resulting in unsatisfactory therapeutic results [3,4].

Traditional prognostic models (e.g., Cox regression scores, the Meta-Analysis Global Group in Chronic Heart Failure [MAGGIC], and Seattle Heart Failure Model) and risk stratification methods were primarily formulated in HFrEF or all-HF cohorts, while exhibiting suboptimal performance in HFpEF given its heterogeneity and non-linear interactions [5,6,7]. Conventional diagnostic scores such as H2FPEF (Heavy [BMI > 30], Hypertensive [≥2 antihypertensive drugs], Atrial Fibrillation, Pulmonary Hypertension [PASP > 35 mmHg], Elder [age > 60 years], Filling Pressure [E/e′ > 9]) and HFA-PEFF (Heart Failure Association Pre-test Assessment, Echocardiography, Functional Testing, Final Etiology) are primarily diagnostic rather than prognostic tools, further limiting their utility for outcome prediction [4,6]. This highlights the need for more sophisticated approaches to risk assessment [6,7]. One of the modern approaches, with growing interest, is machine learning (ML), which can integrate high-dimensional data and capture complex patterns to enhance risk stratification in HFpEF.

In recent years, ML techniques and data-driven approaches have become more common for studying complex data structures and providing crucial insights that regular statistical methods cannot model. ML is pivotal for variable importance, feature extraction, and understanding variable interaction patterns, which enhance data modeling. Another characteristic is its ability to integrate diverse data sources, including electronic health records (EHRs), imaging, biomarkers, and genetics, combined with complex analytical approaches such as decision trees, random forests, gradient boosting, and neural networks. This enables more precise identification of at-risk subgroups and predictors. Prior studies suggest that ML may outperform conventional models in predicting outcomes, such as those for HFpEF, and uncover novel knowledge [8,9,10].

This review was conducted by searching PubMed, Scopus, and Web of Science for English-language articles published between January 2015 and March 2026. Search terms included: (“heart failure with preserved ejection fraction” OR “HFpEF”) AND (“machine learning” OR “random forest” OR “gradient boosting” OR “XGBoost” OR “support vector machine” OR “neural network” OR “deep learning” OR “artificial intelligence”) AND (“risk stratification” OR “prediction” OR “prognosis” OR “mortality”). Studies were included if they: (1) focused on HFpEF populations defined by LVEF ≥ 50%; (2) applied ML algorithms for outcome prediction (mortality, hospitalization, or composite endpoints); (3) reported quantitative performance metrics (area under the curve [AUC], C-statistic, accuracy, or calibration measures); and (4) were original research articles or validated secondary analyses of clinical trials/registries. Exclusion criteria included editorials, case reports, studies with fewer than 50 patients, and ML models developed exclusively for diagnostic (rather than prognostic) purposes. Of the 347 initial records, 28 studies met the inclusion criteria and are discussed in this review.

This review summarizes and evaluates the applications of ML in risk assessment for HFpEF patients, investigating how these sophisticated computational methodologies can enhance predictive accuracy, uncover novel abnormalities, and potentially inform individualized therapy strategies.

## 2. Pathophysiology and Challenges in Risk Assessment of HFpEF

### 2.1. Pathophysiological Mechanisms of HFpEF

The pathophysiology of HFpEF is complex and multifactorial, involving several interrelated mechanisms that culminate in HF while preserving ejection fraction. Unlike HFrEF, which primarily results from systolic dysfunction [11], HFpEF is dominated by diastolic dysfunction—characterized by impaired left ventricular relaxation, increased myocardial stiffness, and elevated filling pressures [11]. At the cellular level, cardiac myocytes in HFpEF are hypertrophied and shortened, with increased collagen deposition that reduces myocardial compliance. Additionally, reduced myocardial capillary density further compromises cardiac performance [12].

Molecular contributors to diastolic failure include hypophosphorylation of titin, which is a key sarcomeric protein that governs cardiomyocyte passive tension. This alteration, driven by reduced cyclic guanosine monophosphate (cGMP), heightens myocyte stiffness [11]. Moreover, disturbances in calcium management and acto-myosin kinetics hinder cross-bridge deactivation, exacerbating diastolic dysfunction [12].

HFpEF is now recognized as a systemic disease with multiple interacting mechanisms. Microvascular dysfunction and endothelial impairment play pivotal roles. According to the “Paulus paradigm,” cardiovascular risk factors trigger systemic inflammation, leading to endothelial and microvascular dysfunction, myocardial ischemia, fibrosis, and diastolic failure [13]. Inflammatory and metabolic derangements—particularly obesity-related inflammation, insulin resistance, and impaired myocardial energetics—further exacerbate HFpEF progression [14,15].

### 2.2. Limitations of Conventional Risk Assessment Tools

Conventional risk assessment tools in HFpEF present notable limitations, complicating both clinical management and research. Existing scoring systems, i.e., H2FPEF (Heavy [BMI > 30 kg/m^2^], Hypertensive [≥2 antihypertensive drugs], Atrial Fibrillation, Pulmonary Hypertension [PASP > 35 mmHg], Elder [age > 60 years], Filling Pressure [E/e′ > 9]) and HFA-PEFF (Heart Failure Association Pre-test Assessment, Echocardiography, Functional Testing, Final Etiology), often show variable performance across populations and inconsistencies in risk classification [6].

The H2FPEF score includes six parameters as defined above. Despite its diagnostic utility, the higher weighting of atrial fibrillation (3 points) and BMI (2 points) may restrict its generalizability [6]. Conversely, the HFA-PEFF score, developed by the European Society of Cardiology (ESC), integrates natriuretic peptide levels with functional and structural echocardiographic markers. While comprehensive, it is complex for routine use and performs variably among patient subgroups [4]. A major drawback of both models lies in their reliance on limited clinical and echocardiographic variables, which fail to encompass the full spectrum of HFpEF pathophysiology [4,6]. Moreover, they are primarily diagnostic tools rather than prognostic instruments, thus limiting their utility for outcome prediction or treatment guidance [4,6].

Generally speaking, biomarker-based methodologies, especially those employing natriuretic peptides such as N-terminal pro-B-type natriuretic peptide (NT-proBNP), are suboptimal in HFpEF. Although elevated peptide levels predict adverse outcomes, many HFpEF patients exhibit normal or mildly increased levels, particularly in obesity, where epicardial adiposity and pericardial constraint affect peptide secretion [16].

These challenges highlight the need for advanced, multidimensional risk-stratification models to integrate heterogeneous data and enhance prediction accuracy and patient classification.

## 3. Overview of Machine Learning in Cardiovascular Medicine

### 3.1. Basic Principles of Machine Learning

ML differs from traditional statistics by learning patterns directly from data rather than relying on predefined models. It enables prediction, classification, and clustering through iterative improvement based on experience. Learning can be supervised, unsupervised, or reinforcement-based [17]. In cardiovascular medicine, supervised and unsupervised approaches are most frequently applied for diagnostic, prognostic, and therapeutic purposes. Algorithms such as logistic regression, random forests, support vector machines, and neural networks use labeled datasets to predict outcomes or classify patients according to clinical, imaging, and biomarker inputs [18]. Unsupervised methods, including clustering and dimensionality reduction, uncover latent patterns and patient subgroups with distinct therapeutic responses [19]. Deep learning, particularly convolutional neural networks, processes complex imaging data from echocardiography and cardiac MRI with high precision [20,21,22]. Algorithm performance is evaluated using accuracy, sensitivity, specificity, AUC, and cross-validation to ensure robustness and generalizability [23].

### 3.2. Machine Learning Applications in Cardiology

ML has revolutionized cardiology by transforming disease diagnosis, risk assessment, therapy selection, and outcome prediction [24]. These applications further enhance ML’s capacity to interpret intricate, diverse data sources, such as EHRs, imaging studies, genetic data, and physiological measures [25].

In cardiac imaging, ML automates measurement, improves image quality, and extracts diagnostic features with high accuracy. Deep learning, particularly in echocardiography, enables automated chamber segmentation, image classification, and quantification of cardiac function. EchoNet, for example, achieved strong performance in detecting pacemaker leads (AUC = 0.89), left atrial enlargement (AUC = 0.86), and left ventricular hypertrophy (AUC = 0.75), while accurately estimating ventricular volumes and ejection fraction [22]. Comparable results have been observed in cardiac MRI, where ML models show excellent agreement with expert-derived measurements [26]. In biomarker-based prediction, ML improves risk estimation by combining troponin, NT-proBNP, and clinical variables, thereby capturing broader pathophysiologic profiles [27]. Predictive modeling for outcomes represents another key domain, with ML algorithms consistently outperforming traditional tools like the Framingham Risk Score [25]. A study comparing logistic regression with ensemble ML models (random forest, XGBoost, deep learning) found improved discrimination for heart disease prediction (AUC = 0.760 vs. 0.737) [28]. By enabling real-time, data-driven decision support, ML significantly advances cardiovascular care, improving diagnostic precision, risk stratification, and individualized treatment planning.

## 4. Machine Learning for Risk Assessment in HFpEF Patients

### 4.1. Data Sources for Machine Learning Models

The development of robust ML models for HFpEF risk stratification depends on data quality, diversity, and comprehensiveness. These datasets span clinical, biological, imaging, and molecular domains, reflecting diverse aspects of the disease [25]. Table 1 summarizes the key data modalities and their important features for ML-based HFpEF risk assessment, including continuous ECG data, echocardiography, cardiac MRI, laboratory data, and clinical EHR data.

Table 2 lists publicly available datasets commonly used for ML-based HFpEF research, including clinical trials, ICU databases, population cohorts, and regional registries.

#### 4.1.1. Electronic Health Records (EHRs)

EHRs are essential for ML model development, offering demographic, clinical, and laboratory information that captures longitudinal disease dynamics [25]. The longitudinal nature of EHR data is particularly valuable, enabling the analysis of disease progression and treatment response over time. Woolley et al. [32] used 363 biomarkers from 429 HFpEF patients to define four prognostically distinct clusters. Likewise, Chang et al. [30] applied a random survival forest model to 6092 HFpEF cases, achieving accurate prediction of hospitalizations and cardiovascular death.

#### 4.1.2. Biomarkers

Traditional cardiac biomarkers such as NT-proBNP and high-sensitivity cardiac troponin T indicate myocardial stress and injury, respectively. Newer biomarkers, including those focusing on inflammation (e.g., tumor necrosis factorα [TNFα], growth/differentiation factor-15 [GDF-15]), extracellular matrix turnover (e.g., tissue inhibitor of metalloproteinase-1 [TIMP-1], matrix metalloproteinases-2 [MMP-2], MMP-9), and endothelial function (e.g., endoglin), provide insights into additional pathophysiological dimensions [16]. A study by Gao et al. [16] combined 18 biomarkers and clinical indicators via a support vector machine, outperforming individual markers in forecasting two-year mortality.

#### 4.1.3. Cardiac Imaging

Echocardiographic indices, including left atrial volume index, E/e’ ratio, global longitudinal strain, and tricuspid regurgitation velocity, provide information about diastolic function, filling pressures, and pulmonary hemodynamics. Cardiac MRI offers supplementary insights into tissue characterization, encompassing the identification and degree of myocardial fibrosis, which may have prognostic significance in HFpEF [26]. ML in cardiac imaging automates analysis and feature extraction, improving diagnostic and prognostic accuracy. Deep learning detects subtle HFpEF patterns, often surpassing human interpretation [21].

#### 4.1.4. Genetic Data

Genetics and omics constitute a new area of research in HFpEF risk stratification. Genomic, transcriptomic, proteomic, and metabolomic investigations elucidate the molecular foundations of HFpEF, potentially uncovering novel biomarkers and therapeutic targets [33]. A study by Jani et al. [15] identified proteomic profiles in HFpEF linked to metabolic dysfunction and impaired translation, particularly in obesity. Integrating proteomic, transcriptomic, and clinical data via ML improved comprehensive risk stratification and personalized therapeutic targeting.

#### 4.1.5. Clinical Trials and Observational Cohorts

Trials like TOPCAT [34] and PARAGON [35] have provided well-characterized HFpEF cohorts for phenotypic analysis. Although trial cohorts are selected, they offer complete follow-up and standardized data. Angraal et al. [34] used TOPCAT data for ML outcome models. Similarly, multi-center observational cohorts can serve as training or external test sets.

#### 4.1.6. Wearables and Remote Monitoring

While not yet widely reported in the HFpEF ML literature, continuous physiological data (activity monitors, implantable hemodynamic sensors, and smartphone echocardiography) offer future data streams. For example, combining home weight/heart rate logs with ML could predict decompensation, but validation in HFpEF populations is needed [29]. As shown in Table 1, continuous ECG data features such as heart rate variability and atrial fibrillation burden are particularly relevant for wearable-derived prediction models.

A recent study by Deng et al. [36] developed a random forest model to predict 1-year readmission in HFpEF patients with concomitant chronic kidney disease (CKD), achieving an AUC of 0.837 (95% CI 0.761–0.905) in temporal validation, significantly outperforming the traditional MAGGIC score (AUC 0.551). The study identified estimated glomerular filtration rate (eGFR) as the primary predictor, with an important interaction between high-sensitivity C-reactive protein (hs-CRP) and NT-proBNP, highlighting the value of ML for high-risk comorbid subgroups [36].

#### 4.1.7. Feature Engineering

Feature engineering is essential in ML modeling to optimize data representation and improve model performance. Continuous variables, such as age or BNP levels, are standardized or discretized, while categorical variables (e.g., comorbidities, medications) are encoded using one-hot encoding or factorization. Dimensionality reduction methods like principal component analysis and autoencoders uncover hidden structures, while domain expertise supports creating composite variables such as comorbidity indices or echocardiographic scores. In high-dimensional datasets, feature selection techniques—including univariate filtering, recursive elimination, and regularization—enhance generalizability and prevent overfitting. Hu et al. [31] identified 53 clinically relevant features, and Zhou et al. [7] reduced genomic variables, improving reproducibility and interpretability.

## 5. Model Development and Evaluation

Developing a robust ML model for HFpEF risk stratification requires a structured process encompassing algorithm selection, training, validation, and interpretability.

### 5.1. Algorithm Choice

Both traditional and advanced algorithms are used. Linear models such as logistic regression and LASSO provide interpretable baselines but often fail to capture HFpEF’s nonlinear complexity. Tree-based ensemble methods (random forest, gradient boosting, XGBoost, and LightGBM) are preferred for handling tabular data and modeling variable interactions. Random survival forests further extend applicability to censored outcomes. Neural networks are less common due to small HFpEF sample sizes, but are effective in imaging applications and may gain relevance with larger datasets. Hybrid or stacking ensembles can further enhance predictive accuracy by integrating multiple models [37].

### 5.2. Training and Validation

Rigorous validation is critical to avoid overfitting. Standard practice includes train–test splits (e.g., 80:20) with cross-validation on the training set. Hu et al. [31] performed 5-fold cross-validation, while Zhou et al. [7] used 1000 random splits to confirm stability. External validation, as demonstrated by Hu et al. [31] with a LightGBM model (AUC = 0.87), is ideal for confirming generalizability.

### 5.3. Performance Metrics

Discrimination is typically evaluated via AUC or C-statistics, while precision–recall curves are valuable for imbalanced outcomes. Calibration, often underreported, assesses agreement between predicted and observed risks. Hu et al. [31] reported AUC, accuracy, sensitivity, specificity, F1, and Brier scores. Confidence intervals obtained by bootstrapping or cross-validation improve robustness. The TRIPOD (Transparent Reporting of a Multivariable Prediction Model for Individual Prognosis or Diagnosis) guidelines advocate transparent reporting of both model development and validation [38].

### 5.4. Interpretability

Interpretability remains a major challenge for black-box ML models. Contemporary ML research emphasizes explainability, with many HFpEF studies employing feature importance rankings or Shapley values (SHAP: SHapley Additive exPlanations) to identify key predictors. For example, SHAP has been applied to interpret XGBoost models [39]. Chang et al. [30] used partial dependence plots to illustrate the impact of individual risk factors on predicted hazard. To enhance clinical usability, some studies translate ML outputs into simplified scoring systems such as nomograms or decision rules. Alternatively, inherently interpretable models, like decision trees or parsimonious logistic regression, may be preferred when predictive performance is adequate [40].

### 5.5. Overfitting and Bias

Overfitting risks arise in high-dimensional, limited datasets. Regularization (e.g., LASSO), early stopping, or reduced model complexity can mitigate this. Feature selection, as employed by Zhou et al. [7], can help minimize noise. Class imbalance, such as low event rates, may be addressed through resampling techniques like SMOTE or cost-sensitive learning. Finally, model bias is also a key concern [41]. For instance, underrepresentation of specific subgroups (e.g., rural or minority populations) can lead to reduced predictive accuracy. Reporting subgroup-specific AUCs by age, sex, or race, and adapting models to diverse populations, when necessary, is recommended [42].

## 6. Machine Learning Algorithms Used in HFpEF Risk Prediction

A variety of ML algorithms have been applied to HFpEF risk prediction, each offering unique advantages, limitations, and domains of suitability. The choice of algorithm depends on data characteristics, prediction goals, and the desired balance between interpretability and predictive performance [7].

### 6.1. Logistic Regression

Logistic regression remains valuable for ML-based risk prediction, particularly when combined with regularization techniques such as elastic net or LASSO [28]. These methods reduce overfitting and enable feature selection in high-dimensional datasets. A study found that logistic regression with elastic net outperformed traditional approaches for predicting cardiovascular risk [28].

### 6.2. Advanced ML Models

Advanced ML models, such as random forests, gradient boosting machines (e.g., XGBoost), and support vector machines, have outperformed standard statistical methods in predicting HFpEF risk. These algorithms can detect intricate, non-linear correlations between predictors and outcomes, thereby uncovering subtle patterns that traditional methods may overlook [28]. A study by Chang et al. [30] utilized a random survival forest model to identify 15 predictors for HF hospitalizations and cardiovascular-related death in HFpEF patients, achieving an AUC of 85.6% and 86.9% in the derivation and validation sets, respectively. Similarly, Xi et al. [28] found that an ensemble model combining random forest, XGBoost, and deep learning outperformed traditional logistic regression in cardiovascular risk prediction.

Innovative noninvasive approaches are also emerging. Yang et al. [43] combined oral hyperspectral imaging with 28 machine learning algorithms to diagnose HFpEF, with random forest achieving an AUC of 0.884 in internal validation and 0.812 in external validation. SHAP analysis identified 25 key spectral and textural features distinguishing HFpEF patients from controls, demonstrating the potential of digital, noninvasive diagnostics [43].

### 6.3. Deep Learning

Deep learning architectures, notably CNNs and recurrent neural networks (RNNs), excel at processing complex, high-dimensional data such as cardiac imaging and longitudinal clinical sequences [44]. These models autonomously learn hierarchical representations, enabling the discovery of novel predictors of adverse outcomes [44]. Deep learning on echocardiography identifies cardiac structures, evaluates function, and predicts systemic phenotypes influencing cardiovascular risk beyond human detection [22].

Beyond imaging applications, Hong et al. [45] developed an artificial intelligence-enabled electrocardiogram (AI-ECG) model using a convolutional neural network to predict HFpEF (defined by HFA-PEFF score ≥ 5) in 13,081 patients, achieving an AUC of 0.81 (95% CI 0.79–0.82). Patients with a positive AI-ECG classification had significantly worse outcomes, including a 9.56-fold higher risk of cardiac death and 5.91-fold higher risk of HF hospitalization at 5 years, demonstrating the prognostic value of ECG-based ML [45].

### 6.4. Unsupervised Learning

Unsupervised learning methods, including clustering algorithms and dimensionality reduction techniques, enable hidden heterogeneity to be revealed among HFpEF patients by analyzing unlabeled data to identify patient subgroups with distinct risks and responses. Shah et al. applied unsupervised ML to extensive phenotypic datasets, identifying three distinct HFpEF phenogroups with significant differences in cardiac structure, hemodynamics, and outcomes. This “phenomapping” approach highlighted HFpEF as a heterogeneous syndrome and demonstrated the potential of ML in uncovering clinically meaningful patient subtypes [3].

Kernel-based algorithms further advance unsupervised modeling. Zhou et al. [7] developed a genetic algorithm-optimized kernel partial least squares (GA-KPLS) model using gene expression data to predict 3-year mortality in HFpEF patients, outperforming models such as random forest, LASSO, ridge regression, support vector machine, and logistic regression.

In clinical applications, interpretability remains essential. While models like decision trees and logistic regression offer transparency, complex architectures like deep neural networks are less intuitive. Explainability techniques, such as SHAP, Local Interpretable Model-Agnostic Explanations (LIME), and attention mechanisms, can elucidate decision processes, improve clinician confidence and facilitate regulatory acceptance [46]. Table 3 summarizes the strengths, weaknesses, and optimal use cases for each ML algorithm discussed above.

## 7. Performance of Machine Learning Models in Risk Stratification

ML algorithms outperformed traditional methods in HFpEF risk stratification, exhibiting greater discrimination, calibration, and reclassification metrics. Evaluation typically involves comparison with standard risk scores and assessment of predictive ability for outcomes such as mortality, hospitalization, and disease progression [48].

Discrimination, measured by the AUC, reflects a model’s capacity to differentiate between patients with and without adverse outcomes. ML models generally achieve higher AUCs than conventional approaches [48]. Table 4 summarizes the performance characteristics of key ML models for HFpEF risk stratification, including discrimination metrics, validation approaches, and key predictors.

The GA-KPLS model created by Zhou et al. [7] to predict 3-year mortality in HFpEF patients had an AUC of 0.955, which was much better than other ML algorithms and traditional statistical methods. Similarly, Chang et al. [30] demonstrated that a random survival forest achieved AUCs of 85.6% and 86.9% for HF hospitalizations and cardiovascular deaths in derivation and validation cohorts, respectively. These findings confirm ML’s ability to accurately identify high-risk HFpEF patients who may benefit from more intensive surveillance and targeted therapy.

Calibration, which measures the agreement between predicted and observed outcomes, is a crucial component of ML model evaluation. A well-calibrated model provides accurate and consistent risk estimates across all probability levels, thereby enhancing clinical reliability and individual risk assessment. Studies evaluating ML approaches for cardiovascular risk prediction have generally reported favorable calibration, especially for ensemble models integrating multiple algorithms. Li et al. [25] demonstrated that XGBoost achieved excellent calibration in predicting cardiovascular risk among men (Dx = 0.598, *p* = 0.75) and women (D = 1.867, *p* = 0.08), confirming reliable predictions across diverse subgroups. This level of calibration ensures that estimated probabilities correspond closely to actual outcomes, strengthening clinical decision-making.

Reclassification metrics, including Net Reclassification Improvement (NRI) and Integrated Discrimination Improvement (IDI), evaluate how effectively models reassign patients into more accurate risk categories compared with reference models. In their cardiovascular risk prediction study, Li et al. [25] demonstrated that ML algorithms such as XGBoost and LASSO achieved notable improvements over traditional Cox models, enhancing reclassification by 3.9% (1.4–6.4%) and 2.8% (0.7–4.9%), respectively, thereby reinforcing their clinical applicability.

ML-based biomarker models have also improved HFpEF risk classification. Gao et al. [16] used a support vector machine integrating 18 biomarkers to predict 2-year all-cause mortality, achieving AUCs of 0.834 and 0.798 for training and validation sets, significantly improving classification performance. Likewise, Shah et al. [3] showed that ML-based phenogrouping provided superior stratification, with phenogroup 3 exhibiting a fourfold higher HF hospitalization risk (hazard ratio = 4.2; 95% CI, 2.0–9.1).

Angraal et al. [34] utilized data from the TOPCAT trial to develop ML models predicting 3-year all-cause mortality and HF hospitalization in HFpEF. They compared logistic regression, LASSO, random forest, gradient boosting, and support vector machine models using 5-fold cross-validation. Among these, the random forest achieved the highest performance, with mean C-statistics of approximately 0.72 for mortality and 0.76 for HF hospitalization. Key predictors included patient-reported health status (Kansas City Cardiomyopathy Questionnaire), renal function, and biomarkers such as blood urea nitrogen and NT-proBNP. Although the improvement over traditional models was modest, the study emphasized the predictive importance of functional status data. Chang et al. [30] analyzed 6092 HFpEF patients using a random survival forest to predict a composite outcome of HF hospitalization or cardiovascular death. From 58 variables, 15 emerged as key predictors, including age ≥ 65, BNP ≥ 600 pg/mL, left atrial diameter ≥ 46 mm, and atrial fibrillation, yielding a time-dependent AUC of 0.869 in external validation. Partial dependence plots enhanced interpretability.

Similarly, Hu et al. [31] developed a LightGBM model predicting 1-year readmission in HFpEF (*n* = 766, 53 variables), achieving AUCs of 0.88 (internal) and 0.87 (external). SHAP analysis identified top features, and a dynamic nomogram translated results into practical clinical scoring.

Wang et al. [47] investigated premature myocardial infarction patients (age < 55) to predict in-hospital HFpEF onset using five ML algorithms: LASSO-logistic regression, XGBoost, random forest, K-nearest neighbors, and support vector machine. Among 840 patients, 32% developed HFpEF, and XGBoost achieved the best performance (AUC = 0.854; accuracy = 0.798). Key predictors included BNP, SYNTAX score, age, inflammation markers, and hypertension. Model interpretability was achieved using SHAP values, and a Shiny web app was developed for bedside risk estimation, illustrating explainable ML in a specialized post-myocardial infarction HFpEF setting.

Ward et al. [49] extended ML applications to molecular data, employing an ensemble algorithm to classify patients as LV hypertrophy or HFpEF using demographics and extracellular matrix biomarkers. The model achieved an AUC of 0.90 for HFpEF diagnosis, even without echocardiography, demonstrating the promise of multi-marker blood-based ML screening. Similarly, Zhou et al. [7] applied several algorithms to gene expression data from 149 HFpEF patients, with GA-KPLS yielding the best 3-year survival prediction and identifying 116 prognostically significant genes. Together, these studies highlight ML’s capacity to integrate clinical, imaging, and molecular data for improved HFpEF risk stratification.

## 8. Challenges and Ethical Considerations

The application of ML to HFpEF risk stratification presents multiple technical, clinical, ethical, and regulatory challenges that must be addressed to ensure reliability, fairness, and clinical trust.

### 8.1. Data Bias

A central issue is data bias, which can arise from imbalanced or unrepresentative datasets. Biases linked to race, socioeconomic status, or healthcare access can result in models that underperform regarding certain populations, reinforcing existing disparities. Studies have shown that such biases can compromise AI-driven tools and worsen health inequalities [50]. Mitigating bias requires inclusive, diverse datasets, transparent variable selection, continuous model auditing, and subgroup-specific performance monitoring.

### 8.2. Model Generalizability

Another major concern involves model generalizability. Despite high accuracy in development datasets, ML models often lose performance when applied to new populations or clinical environments. Complex “black-box” architectures further limit clinical acceptance. Interpretability techniques such as SHAP, LIME, and attention mechanisms can elucidate predictive drivers, while methods like transfer learning, domain adaptation, and federated learning enhance adaptability across healthcare systems.

Addressing privacy concerns in multi-center ML, Liu et al. [51] introduced PerFed-Cardio, a semi-federated learning framework for multimodal cardiac imaging and risk stratification that achieved an AUC of 0.972 while reducing communication load by 28% and preserving data privacy across institutions [51].

### 8.3. **Clinical Interpretability**

Explaining the reasoning behind black-box ML models remains a substantial hurdle. While contemporary ML research stresses the value of interpretability, many HFpEF studies have turned to feature importance rankings or SHAP to isolate key predictive variables.However, recent evidence suggests that SHAP explanations do not consistently improve clinician decision-making and may create false reassurance; human–AI team training is required [39,40].

### 8.4. Privacy and Consent

Ethical considerations around privacy, consent, and data ownership are equally crucial. ML development depends on large-scale patient data, necessitating strict adherence to privacy standards and regulations. Patients should be informed about data usage, and robust cybersecurity measures must protect sensitive information, requiring transparent governance policies defining data ownership and control [52].

### 8.5. Regulatory Oversight

Regulatory oversight remains an evolving domain. Agencies such as the U.S. Food and Drug Administration (FDA) are developing frameworks to evaluate ML-based medical devices and ensure safety, efficacy, and accountability [53]. However, clinical validation remains the ultimate test of model utility. Beyond technical accuracy, models must demonstrate improved clinical outcomes through rigorous studies, ideally randomized controlled trials. Such trials should assess the impact of ML integration on decision-making, patient well-being, and healthcare costs. Innovative validation methods, including AI-driven simulation techniques, may complement traditional trials by replicating real-world patient variability and care pathways [54].

### 8.6. Implementation Barriers and the Clinician–AI Knowledge Gap

Even when ML models demonstrate technical validity, their clinical adoption faces major barriers. First, deficiencies in routine clinical data—missing values, non-standardized variable definitions, and variable recording frequency—degrade model performance when moving from research to real-world settings. Second, a critical knowledge gap exists: clinicians familiar with HFpEF pathophysiology may lack training in ML interpretation, while data scientists may not appreciate clinical nuances. This disconnect prevents effective translation of model outputs into actionable treatment decisions [40]. Third, different data sources (EHR, imaging, wearable devices) often lack interoperability, requiring manual data aggregation.

Proposed solutions include: (1) embedding ML risk scores directly into existing EHR workflows with visual explanations (SHAP summary plots); (2) developing hybrid models that combine ML predictions with rule-based clinical criteria to enhance trust; (3) creating standardized data collection protocols for HFpEF registries; and (4) implementing clinician–AI team training programs. Until these implementation science challenges are addressed, even the most accurate ML models will remain underutilized.

### 8.7. Time-Varying Medication Effects and Treatment Confounding

An additional challenge is that medication use (e.g., beta-blockers, ACE inhibitors/ARBs, MRAs, loop diuretics, and SGLT2 inhibitors) is often incorporated as a covariate in ML models, but treatment effects may change over time or differ across HFpEF phenogroups. For example, SGLT2 inhibitors (empagliflozin and dapagliflozin) have recently shown benefit in HFpEF regardless of diabetes status, yet most existing ML models were developed using pre-SGLT2 inhibitor era data [35]. Future ML models should account for guideline-directed medical therapy as time-varying covariates and be recalibrated as new therapies emerge.

## 9. Clinical Relevance

### Integration into Clinical Practice

**Figure 1:**
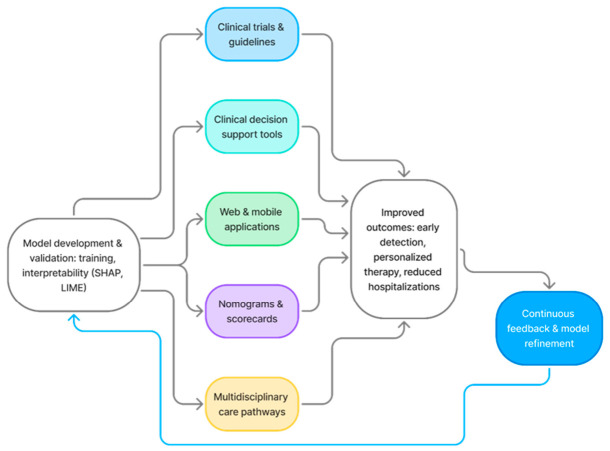
Integration of ML-based risk prediction into clinical practice for HFpEF.

Translating ML models into patient care requires deliberate integration with clinicians’ workflow and decision-making. Figure 1 summarizes key strategies to operationalize ML-based HFpEF risk prediction, including models’ integration into electronic health records as decision support tools, using web and mobile applications for real-time risk estimation, and converting algorithms into simplified nomograms or scorecards for bedside use. ML-guided stratification within multidisciplinary care pathways can personalize monitoring and therapy, potentially reducing adverse events. Finally, incorporating validated ML tools into clinical trials and practice guidelines will accelerate regulatory acceptance and promote evidence-based adoption, bridging the gap between data-driven modeling and everyday clinical management of HFpEF. Continuous feedback from real-world data allows these models to be periodically recalibrated and refined, ensuring sustained accuracy and clinical relevance over time.

While current ML models for HFpEF focus primarily on mortality and hospitalization prediction, emerging applications are addressing procedural risk assessment. ML could potentially guide decisions regarding invasive hemodynamic monitoring, pacemaker implantation for chronotropic incompetence, or evaluation for mitral valve intervention (e.g., TEER) in selected HFpEF patients with secondary mitral regurgitation. However, prospective validation in these specific procedural contexts is lacking, and no ML model for HFpEF has yet been prospectively tested to guide device therapy or surgical referral. This represents an important future direction [9,10].

## 10. Future Directions

The field of ML in HFpEF is advancing rapidly, opening new opportunities to refine prediction and personalize management:

Integrating multi-modal and longitudinal data, including imaging, genomics, proteomics, and wearable monitoring, will enrich risk modeling and capture early disease dynamics. Deep learning applied to sequential ECG or imaging data and federated learning across centers may enhance predictive power while maintaining data privacy.

Rigorous prospective and real-world validation is essential to confirm clinical utility. Embedding ML-based risk tools into HF clinics will enable outcome tracking and continuous model refinement. Progress in explainable and personalized AI, such as counterfactual reasoning and digital twins, will strengthen transparency and patient-specific adaptation.

The expansion of telemedicine and digital health provides new data streams for remote monitoring and predictive alerts, supporting proactive management. Ethical and regulatory frameworks that ensure fairness, accountability, and sustained oversight through multidisciplinary collaboration remain equally important.

Looking ahead, interventional ML that links prediction to tailored therapeutic actions may transform HFpEF care, while trials comparing ML-assisted and standard management will clarify clinical benefit. Ultimately, the goal is a machine–human synergy in which ML augments rather than replaces clinical judgment.

Enhancing AI literacy among clinicians will be essential to ensure responsible adoption and improved patient outcomes.

Beyond traditional ML, agentic AI—autonomous systems that can plan, execute, and refine actions based on real-time data—is emerging in cardiovascular care. While no published study has applied agentic AI specifically to HFpEF risk stratification as of March 2026, relevant precedents include AI agents for dynamic medication titration in heart failure (e.g., autonomous diuretic adjustment based on weight and creatinine trends) and multi-agent systems for coordinating remote monitoring alerts with clinical workflows [29]. Future agentic AI systems for HFpEF could: (1) continuously integrate wearable data, EHR updates, and patient-reported outcomes; (2) trigger automated risk reassessments when new data deviate from predicted trajectories; and (3) propose personalized care plans (e.g., intensifying monitoring, scheduling echo, adjusting diuretics) for clinician approval. Prospective validation of such agentic approaches remains a critical research gap.

A comprehensive 2026 review by Yi et al. [55] synthesizes current AI applications in HFpEF across diagnosis, phenotyping, risk stratification, and management, emphasizing the need for prospective validation and clinical workflow integration as the next critical steps for the field [55].

## 11. Conclusions

HFpEF remains a major clinical and public health challenge due to its heterogeneity and limited therapeutic options. Conventional risk stratification fails to capture this complexity, whereas ML enables superior prediction and individualized assessment by integrating multidimensional data, including clinical, biomarker, imaging, and omics inputs. Beyond outcome prediction, ML has revealed distinct HFpEF phenotypes with therapeutic implications. Yet, challenges related to data bias, interpretability, validation, and regulation persist. Future progress requires multidisciplinary collaboration, robust validation, and patient-centered implementation to ensure safe integration, optimize outcomes, and advance precision management in HFpEF.

## Figures and Tables

**Table 1:** Key data modalities and important features for ML-based HFpEF risk stratification.

Modality	Important Features for HFpEF Risk Assessment	Example ML Application
Continuous ECG data	Heart rate variability, nocturnal heart rate, atrial fibrillation burden, premature atrial/ventricular contractions	Prediction of decompensation (wearable-derived features) [29]
Echocardiography	E/e’ ratio, left atrial volume index (LAVI), tricuspid regurgitation velocity (TRV), global longitudinal strain (GLS), pulmonary artery systolic pressure (PASP)	Automated feature extraction and outcome prediction [21,22]
Cardiac MRI	Extracellular volume fraction (ECV), late gadolinium enhancement (LGE) for fibrosis, T1 mapping, left atrial strain	Tissue characterization and prognostic phenotyping [26]
Laboratory data	NT-proBNP, high-sensitivity troponin T, GDF-15, TIMP-1, MMP-2/9, endoglin, TNFα, creatinine/BUN, hemoglobin	Multi-biomarker ML models for mortality prediction [16]
Clinical EHR data	Age, BMI, hypertension, diabetes, atrial fibrillation, renal function (eGFR), KCCQ score	Random survival forest and LightGBM models [30,31]

**Table 2:** Publicly available and commonly used datasets for ML in HFpEF.

Dataset Name	Population	Sample Size (HFpEF)	Key Features	Access
TOPCAT	Clinical trial (aldosterone antagonist)	~1767	Echo, biomarkers, KCCQ, outcomes	Limited access (request)
PARAGON-HF	Clinical trial (sacubitril/valsartan)	~4796	Comprehensive echo, NT-proBNP, outcomes	Limited access (request)
MIMIC-IV	ICU database (Beth Israel)	Variable	EHR, labs, vitals, medications	Public (physionet.org)
eICU Collaborative Research Database	Multi-center ICU	Variable	Continuous monitoring and labs	Public (physionet.org)
UK Biobank	Population cohort	~2500	Imaging, genetics, biomarkers	Approved application
Chang Gung Research Database	Taiwan health system (Chang et al., 2024 [30])	6092	EHR, echo, outcomes	Not publicly available
HFpEF Network Registry	Multi-center US/Europe	~1500	Proteomics, clinical, outcomes	Collaborative access

**Table 3:** Strengths and weaknesses of ML algorithms for HFpEF risk stratification.

Algorithm	Strengths	Weaknesses	Suitability for HFpEF
Logistic regression (with regularization)	Highly interpretable, fast, no tuning	Linear assumptions; cannot model complex interactions	Baseline model; good for small datasets
Random forest	Handles non-linearity, feature importance, robust to outliers	Less interpretable; may overfit with high noise	Excellent for tabular EHR data [30]
XGBoost/LightGBM	High accuracy, handles missing data, fast training	Hyperparameter tuning required; black box	Best performing for most HFpEF outcomes [31,47]
Support vector machine	Effective in high dimensions; kernel trick	Poor interpretability; slow with large n	Useful for biomarker panels [16]
Neural networks/deep learning	Learns hierarchical features; excellent for imaging	Requires large data, black box, overfitting risk	Ideal for echo/MRI analysis [22]
GA-KPLS	Handles gene expression data well	Complex, not generalizable, small n only	Exploratory molecular studies [7]
Random survival forest	Handles censored outcomes; time-to-event	Complex; calibration challenges	Best for time-to-event outcomes [30]

**Table 4:** Performance of Machine Learning Models for HFpEF Risk Stratification.

Study (Year)	ML Algorithm	Sample Size (*n*)	Outcome Predicted	AUC/C-Statistic	95% Confidence Interval	Validation Type	Key Predictors
Zhou et al. (2021) [7]	GA-KPLS	149	3-year mortality	0.955	NR	Internal (1000 splits)	116 differentially expressed genes
Chang et al. (2024) [30]	Random Survival Forest	6092	CV death/HF hospitalization	0.869	0.84–0.89	External	Age ≥ 65, BNP ≥ 600 pg/mL, LAVI ≥ 46 mm, AF
Hu et al. (2025) [31]	LightGBM	766	1-year readmission	0.88	0.84–0.91	External	E/e’ ratio, NYHA class, LVEF, age, BNP, AF history
Wang et al. (2025) [47]	XGBoost	840	In-hospital HFpEF (post-MI)	0.854	0.82–0.88	Internal (Cross-validation)	BNP > 100 pg/mL, SYNTAX score > 14.5, Age, MLR
Gao et al. (2021) [16]	SVM	318	2-year all-cause mortality	0.834	0.77–0.90	Internal	NT-proBNP, hs-TnT, GDF-15, TNFα, TIMP-1, MMP-2/9
Angraal et al. (2020) [34]	Random Forest	1767	3-year mortality	~0.72	0.69–0.75	5-fold CV	BUN, BMI, KCCQ score, hemoglobin
Angraal et al. (2020) [34]	Gradient Boosting	1767	3-year HF hospitalization	~0.76	0.71–0.81	5-fold CV	Hemoglobin, BUN, prior HF hospitalization, KCCQ

Abbreviations: AUC, area under the curve; BNP, B-type natriuretic peptide; CV, cross-validation; GA-KPLS, genetic algorithm-optimized kernel partial least squares; HF, heart failure; HFpEF, heart failure with preserved ejection fraction; KCCQ, Kansas City Cardiomyopathy Questionnaire; LAVI, left atrial volume index; LightGBM, light gradient boosting machine; ML, machine learning; MLR, monocyte-to-lymphocyte ratio; NR, not reported; NT-proBNP, N-terminal pro-B-type natriuretic peptide; SVM, support vector machine; XGBoost, extreme gradient boosting.

## Data Availability

No new data were created or analyzed in this study. Data sharing is not applicable to this article.

## Abbreviations

Abbreviation	Full form
ACE	Angiotensin-converting enzyme
AI	Artificial intelligence
ARB	Angiotensin receptor blocker
AUC	Area under the curve
BMI	Body mass index
BNP	B-type natriuretic peptide
BUN	Blood urea nitrogen
cGMP	Cyclic guanosine monophosphate
CNN	Convolutional neural network
CV	Cardiovascular
ECG	Electrocardiogram
ECV	Extracellular volume
eGFR	Estimated glomerular filtration rate
EHR	Electronic health record
ESC	European Society of Cardiology
FDA	Food and Drug Administration
GA-KPLS	Genetic algorithm-optimized kernel partial least squares
GDF-15	Growth/differentiation factor-15
GLS	Global longitudinal strain
H2FPEF	Heavy, Hypertensive, Atrial Fibrillation, Pulmonary Hypertension, Elder, Filling Pressure Score
HF	Heart failure
HFA-PEFF	Heart Failure Association Pre-test Assessment, Echocardiography, Functional Testing, Final Etiology
HFmrEF	Heart failure with mildly reduced ejection fraction
HFpEF	Heart failure with preserved ejection fraction
HFrEF	Heart failure with reduced ejection fraction
IDI	Integrated discrimination improvement
KCCQ	Kansas City Cardiomyopathy Questionnaire
LASSO	Least absolute shrinkage and selection operator
LAVI	Left atrial volume index
LGE	Late gadolinium enhancement
LightGBM	Light gradient boosting machine
LIME	Local interpretable model-agnostic explanations
LVEF	Left ventricular ejection fraction
MAGGIC	Meta-Analysis Global Group in Chronic Heart Failure
ML	Machine learning
MMP	Matrix metalloproteinase
MRA	Mineralocorticoid receptor antagonist
MRI	Magnetic resonance imaging
NRI	Net reclassification improvement
NT-proBNP	N-terminal pro-B-type natriuretic peptide
PASP	Pulmonary artery systolic pressure
RNN	Recurrent neural network
SHAP	SHapley Additive exPlanations
SGLT2	Sodium–glucose cotransporter-2
SMOTE	Synthetic minority oversampling technique
SVM	Support vector machine
TEER	Transcatheter edge-to-edge repair
TIMP	Tissue inhibitor of metalloproteinase
TNFα	Tumor necrosis factor alpha
TOPCAT	Treatment of Preserved Cardiac Function Heart Failure with an Aldosterone Antagonist Trial
TRIPOD	Transparent Reporting of a Multivariable Prediction Model for Individual Prognosis or Diagnosis
TRV	Tricuspid regurgitation velocity
XGBoost	Extreme gradient boosting
